# Role of Nrf2/ARE Pathway in Protective Effect of Electroacupuncture against Endotoxic Shock-Induced Acute Lung Injury in Rabbits

**DOI:** 10.1371/journal.pone.0104924

**Published:** 2014-08-12

**Authors:** Jian-bo Yu, Jia Shi, Li-rong Gong, Shu-an Dong, Yan Xu, Yuan Zhang, Xin-shun Cao, Li-li Wu

**Affiliations:** Department of Anesthesiology, Tianjin Nankai Hospital, Tianjin Medical University, Tianjin, China; Emory University, United States of America

## Abstract

NF-E2 related factor 2 (Nrf2) is a major transcription factor and acts as a key regulator of antioxidant genes to exogenous stimulations. The aim of current study was to determine whether Nrf2/ARE pathway is involved in the protective effect of electroacupuncture on the injured lung in a rabbit model of endotoxic shock**.** A dose of lipopolysaccharide (LPS) 5 mg/kg was administered intravenously to replicate the model of acute lung injury induced by endotoxic shock. Electroacupuncture pretreatment was handled bilaterally at Zusanli and Feishu acupoints for five consecutive days while sham electroacupuncture punctured at non-acupoints. Fourty anesthetized New England male rabbits were randomized into normal control group (group C), LPS group (group L), electroacupuncture + LPS group (group EL) and sham electroacupuncture + LPS (group SEL). At 6 h after LPS administration, the animals were sacrificed and the blood samples were collected for biochemical measurements. The lungs were removed for calculation of wet-to-dry weight ratios (W/D), histopathologic examination, determination of heme oxygenase (HO)-1 protein and mRNA, Nrf2 total and nucleoprotein, as well as Nrf2 mRNA expression, and evaluation of the intracellular distribution of Nrf2 nucleoprotein. LPS caused extensive morphologic lung damage, which was lessened by electroacupuncture treatment. Besides, lung W/D ratios were significantly decreased, the level of malondialdehyde was inhibited, plasma levels of TNF-α and interleukin-6 were decreased, while the activities of superoxide dismutase, glutathione peroxidase and catalase were enhanced in the electroacupucnture treated animals. In addition, electroacupuncture stimulation distinctly increased the expressions of HO-1 and Nrf2 protein including Nrf2 total protein and nucleoprotein as well as mRNA in lung tissue, while these effects were blunted in the sham electroacupuncture group. We concluded that electroacupuncture treatment at ST36 and BL13 effectively attenuates lung injury in a rabbit model of endotoxic shock through activation of Nrf2/ARE pathway and following up-regulation of HO-1 expression.

## Introduction

Pulmonary dysfunction is documented as a hallmark of sepsis, and diffuse lung injury resulting in acute respiratory distress syndrome has been put forward as the major characters of pulmonary dysfunction after endotoxin administration [1]. Intravenous infusion of LPS induced acute lung injury and caused alterations in lung physiologic processes, which was similar to those in humans [2]. However, the precise mechanism involved in the formation of acute lung injury (ALI) or acute respiratory distress syndrome (ARDS) induced by endotoxin is not, as yet, fully understood. Our previous study showed that the increased oxidative stress may be a major cause of organ failure and high mortality during endotoxic shock [3]. Oxidative stress is defined as a condition of imbalance between reactive oxygen species (ROS) formation and cellular antioxidant capacity owing to overproduction of ROS or dysfunction of the antioxidant system [4].Thus, repairing the imbalance status by scavenging ROS or enhancing cellular antioxidant capacity may have implication for a wide array of pathology and disease models.

Acupuncture as an integral part of a traditional Chinese medical system for more than 2500 years. It is worthy of applying acupuncture to specific acupoints to achieve favorable regional or systemic effects [5]. It was proven that electroacupuncture pretreatment significantly inhibited systemic inflammatory responses and improved survival rate in rats with lethal endotoxemia [6]. Feishu (BL13) acupoint is considered of choice to treat lung diseases and regulate pulmonary functions, Pan et al. reported that treatment with electroacupuncture on BL13 showed beneficial effects on hypoxia-induced pulmonary hypertention in rats [7]. Traditionally, acupuncture at Zusanli (ST36) acupoints was known as the modulation of immune functions and is often used in clinical disorders of the immune system [8].

Nuclear factor erythroid-2 related factor-2 (Nrf2) as a Cap “n” Collar basic leucine zipper transcription factor plays a crucial role in regulating antioxidant and cytoprotective genes in response to oxidative stress [9]. An accumulation of ROS or electrophilic compounds give rise to the disruption of Nrf2/Kelch-like ECH-associated protein-1 (Keap1) complex and result in the translocation of Nrf2 from the cytoplasm into the nucleus where it dimerizes with antioxidant response element (ARE) DNA sequence ultimately activates the expression of ARE-dependent genes [10]. Downstream targets of Nrf2 include direct antioxidant proteins such as catalase (CAT) and glutathione peroxidase (GPx), stress-response proteins such as HO-1 and phase II metabolizing enzymes such as glutathione S-transferase (GST), and others [11]. Among these, CAT and GPx directly neutralize ROS and are regarded as very important antioxidant enzymes [4]. Heme oxygenases (HO-1), and with the productions of heme catabolism including biliverdin, bilirubin, carbon monoxide (CO) andiron, shows anti-inflammatory and antioxidant properties [12]. Our preliminary studies have confirmed that up-regulation of HO-1 protein followed by CO increasing could lessen the mortality in septic shock rats [13]. Another previous study elucidated the protective effects of electroacupuncture stimulation at ST36 and BL13 acupoints against acute lung injury evoked by endotoxic shock in rabbits were dependent on up-regulated HO-1 expression [14].

However, it is not known whether electroacupuncture stimulation play a protective role in impaired lung by activating Nrf2/ARE pathway during endotoxic shock. Based on these previous data, we hypothesized that electroacupuncture treatment at ST36 and BL13 acupoints protect against endotoxic shock-induced lung injury via modulating Nrf2/ARE pathway.

## Materials and Methods

### Animals

The current study was conducted in accordance with the Institutional Animal Use Guidelines and approved by the Animal Care Committee of Tianjin Nankai Hospital (NKH-20120818, Tianjin, China). Two-month-old male New England white rabbits (1.5∼2.0 kg) was provided by Laboratory Animal Center of Nankai Clinical Institution of Tianjin Medical University. The animals were housed at 18∼22°C on a 12-h light-dark cycle. Besides, food and water were supplied *ad libitum* for a 5-day period prior to the experiment protocols.

Prior to the induction of anesthesia, the rabbits were fasted for 12 h but allowed free access to water. All the rabbits were anesthetized with 20% urethane (5 ml/kg) via the marginal ear vein and anesthesia was maintained with intravenous infusion of ketamine at 3 mg/Kg/h throughout the experiment. Then, a 3.5 mm non-cuffed endotracheal tube was inserted and tied in place through the tracheotomy. Mean arterial pressure was continuously monitored with a PE-50 catheter inserted through the right carotid artery by using Hellige monitor instruments (Germany), while a 24-g catheter was inserted into internal jugular vein for intravenous injections. As Nishina et al. described [15], the lungs of animals were mechanically ventilated with an infant ventilator (IV100B, Sechrist, Anaheim, CA) at an inspired oxygen concentration of 40%. Tidal volume was set to 10 ml/kg (peak inspiratory pressure was 11–13 cm H_2_O), as measured by pneumotachograph, and 2 cm H_2_O of peak expiratory pressure was added [16]. Respiratory rate was controlled to produce initial PaCO_2_ of 35–40 mmHg while the inspiratory/expiratory time ratio was set at 1∶2 [17]. The rabbits were placed on a heating pad under a radiant heat lamp so as to keep the body temperature at 37.7–40.3°C. Lactated Ringer’s solution was administered intravenously at a rate of 8 ml/kg/h.

### Electroacupunture protocols

All animals were lightly immobilized using hands to minimize stress during acupuncture stimulation which was initiated for a five-consecutive-day before the experiment. Besides, electroacupunture stimulation was performed throughout the operating steps for 6 h during the experimental day [18]. The selected acupoints in this study were Zusanli (ST36), located between the tibia and fibular approximately 5 mm lateral to the anterior tubercle of the tibial, and Feishu (BL13), located between T3 and T4 of the spine approximately 1.5 cm lateral to the midline. A set of non-acupoints located on 5 mm lateral to the ST36 or BL13 original location as controls. Two pairs of stainless steel needles (diameter, 0.3 mm) were inserted bilaterally to a depth of 5 mm into the acupoints and kept in place. The parameter of electroacupuncture was applied for 15 minutes with a disperse-dense wave (ie, alternating frequencies of 2 Hz and 15 Hz) once a day by an electrical stimulation device (HANS G6805-1A, Huayi Co, Shanghai, China) [19], and the intensity was adjusted to induce moderate muscle contract of the hindlimb (≤1 mA) [20]. Acupuncture points were identified by an experienced acupuncturist (ZY).

### Experimental design

Forty Rabbits were randomized into four different groups (n = 10/group): group C, group L, group EL and group SEL. Rabbits in group L, EL and SEL were treated with intravenous injection of 0.5 ml (5 mg/kg) LPS (L2630, sigma, USA) to replicate the experimental model of acute lung injury induced by endotoxic shock, while group C received 0.5 ml normal saline intravenously as a control. Electroacupuncture treatment at ST36 and BL13 bilaterally was conducted in group EL from the preparation of the model until the end of the experiment for 6 h. Meanwhile, group SEL was acupunctured at non-acupoints with the same frequency and intensity described as above. There was no electroacupuncture stimulation in group C. Mean arterial blood pressure (MAP) was monitored continuously and recorded at 30, 60, 90 and 120 min after the start of administration of LPS. MAP did not decrease within 2 h or rabbits died within 6 h after LPS administration were regarded as the exclusion criteria of the study.

### Preparation and analysis of samples

The whole blood was withdrawn from the right carotid artery at 6 h after LPS or normal saline administration. Approximately 1 ml of the arterial blood samples were analyzed for the calculation of oxygenation indexes by a blood gas analyzer (Gem premier 3000, USA) before death. Meanwhile, the blood specimen remained were centrifuged at 4°C (3000 rpm for 15 min). The plasma was removed and the aliquots of the supernatant were separately frozen at −80°C for subsequent analysis. At the end of the experiment, the rabbits were sacrificed by exsanguination, and the lungs were removed and quickly flushed with phosphate-buffered saline (PBS) to remove the blood. Ultimately, sections of the left lung tissues were put in 10% formaldehyde for histopathological analysis, and the remaining tissues were snap frozen in liquid nitrogen and stored at −80°C for subsequent analysis.

### Biochemical measurements

The tissue homogenate was prepared for biochemical assays by the upper lobe of the right lung. The levels of superoxide dismutase (SOD) activities and malondialdehyde (MDA) contents in the lung tissues were determined by spectrophotometry [21] and measured by means of Loewenberg [22], respectively. And both were expressed as per unit of protein determined by the Lowry method [23]. Moreover, the serum level of glutathione peroxidase (GPx) and catalase (CAT) activities were measured using commercial assay kits supplied by the Nanjing Jiancheng Bioengineering Institute (Nanjing, China). Plasma tumor necrosis factor-alpha (TNF-α) and interleukin-6 (IL-6) were assayed with a commercial enzyme-linked immunosorbent assay kit (R&D systems, USA). All the procedures were performed according to manufacturer protocols.

### Lung wet-to-dry (W/D) weight ratios

The W/D weight ratio of the lung was calculated to evaluate the severity of pulmonary edema. The harvested tissue of left upper lobe was rinsed with normal sodium to scour off the superfluous water. After that, the lung tissue was weighed as wet weight. Dry weight was recorded after the specimen was dried to a constant weight at 70°C for 24 h in an electric air blast drier. The W/D weight ratio was then calculated [14].

### Preparation of BALF and Measurements

The Bronchoaleveolar lavage fuild (BALF) analysis was measured to quantify the magnitude of the pulmonary edema. 40 ml saline with ethylendiamine tetraacetic acid (EDTA)-2Na at 4°C was slowly infused for 5 times through the right mainstem bronchus and withdrawn. BALF was analyzed for cell differentiation and cell counts by the Bürker-Türk method [2]. Lavage samples were centrifuged at 250 g at 4°C for 10 min to remove the cells. The cell-free supernatant was analyzed for albumin determined by immune-nephelometry.

### Histopathological examination

Immediately after the rabbits were killed (<5 min), the middle lobe of right lung was fixed in 10% formaldehyde for 24 h, and then dehydrated with graded alcohol followed by embedding in paraffin at 60°C. A battery of microsections (4 µm) stained with hematoxylin and eosin were examined under a light microscope (×400) and ten different visual fields were observed for each slice. The lung pathologic change was assessed by alveolar edema, airway congestion, widening of the interstitium or hyaline membrane formation and reactive cell infiltration or aggregation [24]. Each item was graded according to a 5-point scale described as follows [25]: 0 = minimal damage, 1+ = mild damage, 2+ = moderate damage, 3+ = severe damage, and 4+ = maximal damage. The individual scores were added together to rack up a final score ranging from 0 to 16. The lung injury assessment was quantified by a blinded expert pathologist.

### Western blot analysis

The expression of HO-1, Nrf2 total protein and Nrf2 nucleoprotein of the lung samples were analyzed by Western blot technique. The tissues stored at −80°C were homogenized in 13.2 mmol/L Tris-HCl, 5.5%glycerol, 0.44%SDS and 10% β-mercaptoethanol. The proteins were extracted according to the instructions of the total protein and nuclear protein extraction kit (Thermo, USA), while the protein concentration was detected on the basis of the BCA protein assay kit (Thermo, USA). Equal amounts of soluble protein were fractionated by 12% SDS-PAGE and were transferred to a PVDF membrane (Bio-Rad, USA). Blots were washed triple for 5 min in TBS and then were incubated overnight at 4°C with polyclonal rabbit antibodies against HO-1 (1∶800, Abcam, UK) or Nrf2 (1∶300, Biorbyt, UK). Primary antibodies were diluted in blocking solution containing 1% nonfat milk plus 0.5% BSA in TBS-0.05% Tween 20. After three washes with TBS-0.05% Tween 20, blots was incubated at 37°C for 1 h with the horseradish peroxidase (HRP)-conjugated goat anti-rabbit IgG (1∶3000 dilation, Biorbyt, UK). The blots were visualized with the enhanced chemiluminesence (Bio-Rad, USA) according to the manufacturer’s instruction [26], and the relative density of bands was quantified by densitometry (Molecular Analyst Image-analysis Software, Bio-Rad, USA).

### RNA isolation and Real-time PCR

Total RNA was extracted from lung tissue using a Total Quick RNA kit (TA200TQR, Talent, Italy). Tissue was lysed in the provided buffer and RNA was eluted by RNAse-free water. Total amount of RNA was determined by absorbance at 260 nm, while the purity of RNA were measured by 260/280 nm absorbance ratio, respectively. 1 µg total RNA was reversely transcribed into cDNA with random hexamers using a Revert Aid™ First Strand cDNA Synthesis kit (MetaBiosInc, Canada). The final PCR reaction of volume of 20 µl was established with SYBR Green master mix. Predegeneration of the PCR mix was at 95°C for 10 min, and the thermal cycle profile was denaturing for 20 s at 94°C, annealing for 20 s at 59°C and extension for 20 s at 59°C. A total of 40 PCR cycles were used. The primers were as followed: β-actin sense, 5′-CGCGACATCAAGGAGAAGCTG-3′ and β-actin antisense, 5′-ATTGCCAATGGGTGATACCTG-3′, 128 bp; HO-1sense, 5′-TGCCGAGGGTTTTAAGCTGGT-3′ and HO-1 antisense, 5′-AGAAGGCCATGTCCAGCTCCA-3′, 158 bp; Nrf2 sense, 5′-CCCACAAGTTCGGCATCCAC-3′ and Nrf2 antisense, 5′-TGGCGATTCCTCTGGCGTCT-3′, 182 bp. The comparative C_t_ (threshold cycle) method was applied for quantitiation of target gene expression as described by Schmittgen et al [27]. The relative gene expression of HO-1 and Nrf2 mRNA were normalized to that of β-actin.

### Immunoflurescence assay

The intracellular distribution of Nrf2 was displayed by immunofluorescence technique. Paraffin-embedded tissue sections (4 µm) were dewaxed in xylene and rehydrated in graded ethanol solutions. The antigen retrieval method with citric acid solution was proceeded for 5 min at 95°C, and then was washed in PBS. Follwing that, the tissues were permeabilized with 0.5%Triton X-100 for 15 min and blocked in normal goat serum at room temperature for 30 min [28]. After incubation with the polyclonal Nrf2 primary antibody conjugated to FITC (1∶150, Biorbyt, UK) at 4°C overnight, the sections was washed triple with PBS. At last, the nuclei were counterstained with DAPI (Roche, Shanghai, China) and visualized using a fluorescent microscope (Olympus U-25ND25, Tokyo, Japan). The green staining was showed in cytoplasm and nucleus, while blue staining was in nucleus. The overlay color was considered to be positive. Ultimately, the results were evaluated by semi-quantitative analysis based on the proportion of the Nrf2 nucleoprotein to the number of nuclei in five fields of each slices at a 400 multiple signal magnification [29].

### Statistical analysis

Values were expressed in mean±SD or median (range), except for lung injury scores, for which the Kruskal-Wallis rank test was used. Parametric data were analyzed by one-way analysis of variance (ANOVA) and variations of different groups were compared using the Tukey-Kramer post hoc test. Repeated-measures data (eg. PaO_2_/FiO_2_) were determined by repeated-measures ANOVA. The W/D ratios were analyzed by two-way ANOVA followed by Bonferroni correction. Data of real-time PCR were tested by a t test with two-tailed hypothesis testing. SPSS19.0 statistical software was used for data analysis and P<0.05 was deemed as statistically significant.

## Results

### Death

The death rate in group EL (1 rabbits) was lower than that in group L (4 rabbits), and was higher than that in group C (zero rabbits). There was no significant difference in death rate between group SEL (3 rabbits) and group L. And, the animals were supplemented according to the randomized crossover principles.

### Hemodynamic and oxygenation indexes

MAP in rabbits were remained stable throughout the experiment and the baseline MAP of each group were similar (105∼109 mmHg) (table 1). Sixty minutes after LPS injection, MAP in group EL was distinctly lower than that in group C, and higher than that in group L (P<0.05). There was no significant difference between group L and SEL (P>0.05). Oxygenation indexes were decreased to less than 300 mmHg in group L, EL and SEL at the end of LPS administration. However, electroacupuncture treatment, rather than sham electroacupuncture stimulation could attenuate the reduction, which revealed that oxygenation indexes in group EL was higher than group L (P<0.05).

**Table 1:** Changes in MAP and Oxygenation indexes among four groups.

Groups	Baseline MAP(mmHg)	MAP after electro-acupuncture for30 min (mmHg)	Time after the start of LPS or saline				OxygenationIndexes (mmHg)
			30 min	60 min	90 min	120 min	
C	105±18	103±15	106±17	102±19	103±16	104±13	436±42
L	109±26	105±24	95±29*	83±27*	75±21*	62±28*	195±83*
EL	106±23	123±16* +	109±22+	97±24* +	88±28* +	79±19* +	248±59* +
SEL	107±29	110±19	92±27*	84±22*	71±24*	65±22*	189±94*

*Abbreviations*: *MAP*, mean arterial pressure; *LPS*, lipopolysaccharide. Values as means ± SD (n = 10).

*P<*0.05* versus control group;

+ *P*<*0.05* versus LPS group.

### Lung W/D weight ratio

The lung wet-to-dry weight ratio was calculated as an indicator of pulmonary edema. W/D ratio was increased in the rabbits received LPS (Group L, EL and SEL) compared to group C (P<0.05) (Table 2). Electroacupuncture treatment attenuated the increase of W/D weight ratio (attenuation 50.5%, P<0.05), while group SEL did not show the protective effect (P>0.05).

**Table 2:** Comparisons of W/D ratio, MDA contents and SOD activities in the lung tissue, as well as CAT and GPx activities in serum among four groups.

Groups	W/D Ratio	MDA (nmol/mgprotein)	SOD (U/mgprotein)	CAT(U/ml serum)	GPx (U)
C	3.76±0.21	1.96±0.48	88.93±23.76	67.85±15.25	381.37±74.94
L	5.68±0.25*	4.37±0.72*	47.24±12.28*	36.83±12.19*	213.53±61.94*
EL	4.71±0.17* +	3.25±0.52* +	63.48±18.76* +	50.74±19.13* +	303.46±86.29* +
SEL	5.65±0.35*	4.33±0.83*	46.37±13.34*	31.26±11.99*	219.88±70.54*

*Abbreviations*: *W/D,* wet to dry weight ratio; *MDA,* malondialdehyde; *SOD,* superoxide dismutase; *CAT,* catalase; *GPx*, glutathione peroxidase. Values as means ± SD (n = 10).

*P<*0.05* versus control group;

+ *P*<*0.05* versus LPS group.

### MDA contents and SOD activities in the lung tissue

The comparisons of MDA contents and SOD activities were showed in Table 2. Intravenous administration of LPS showed an apparent increase of MDA contents and decrease of SOD activities compared to group C (P<0.05). However, electroacupuncture reduced the contents of MDA by 53.5% and enhanced activities of SOD by 34.3% to counteract the effects induced by LPS (P<0.05). No significant influence in above parameters were discovered when compared group SEL with group L (P>0.05).

### GPx and CAT activities in serum

Our data revealed that rabbits from group L, EL plus group SEL possessed lower GPx and CAT activities than control group (P<0.05) (Table 2). Moreover, the activities of GPx and CAT, which were known as the ROS direct scavengers were enhanced significantly in group EL (augment 30.6% for GPx and 50.4% for CAT) compared with group L or group SEL (P<0.05). There were no significant differences between group SEL and group L (P>0.05).

### Plasma levels of TNF-α and IL-6

As shown in Table 3, plasma levels of TNF-α and IL-6 in group L, EL and SEL were significantly higher than in group C. However, the EL group showed lower levels of TNF-α and IL-6 than the L group (TNF-α, 19.62±4.89 and 26.79±7.65, P<0.05; IL-6, 87.53±16.23 and 112.32±25.76, P<0.05). We did not find a significant difference in plasma levels of TNF-α and IL-6 between group SEL and group L (P>0.05).

**Table 3:** Comparisons of plasma TNF-α and IL-6 levels and analysis of Bronchoalveolar lavage fluid.

Groups	TNF-α (pg/ml)	IL-6 (pg/ml)	Leukocytes(cells/mm^3^)	BALF Albumin (mg/dl)
C	12.17±2.93	26.53±4.45	197±19	1.2±0.2
L	26.79±7.65*	112.32±25.76*	481±34*	10.3±1.4*
EL	19.62±4.89* +	87.53±16.23* +	375±26* +	6.4±0.7* +
SEL	27.18±8.11*	106.58±20.87*	455±31*	9.7±1.1*

*Abbreviations*: *BALF,* Bronchoalveolar lavage fluid; *TNF-α,* tumor necrosis factor-alpha; *IL-6*, interleukin-6. Values as means ± SD (n = 10).

*P<*0.05* versus control group;

+ *P*<*0.05* versus LPS group.

### Analysis of BALF

The recovery percentages of BALF among the four groups were 83%∼88%, indicating no differences in the groups. Compared with group C, the number of leukocytes and albumin concentrations in the supernatant of BALF were obviously higher in group L, EL and SEL (Table 3). However, electroacupuncture treatment mitigated the increase in leukocyte counts and albumin concentrations in the BALF in rabbits receiving LPS. In contrast, there was no significant difference in BALF concentrations between group SEL and group L.

### Histopathological grading

**Figure 1:**
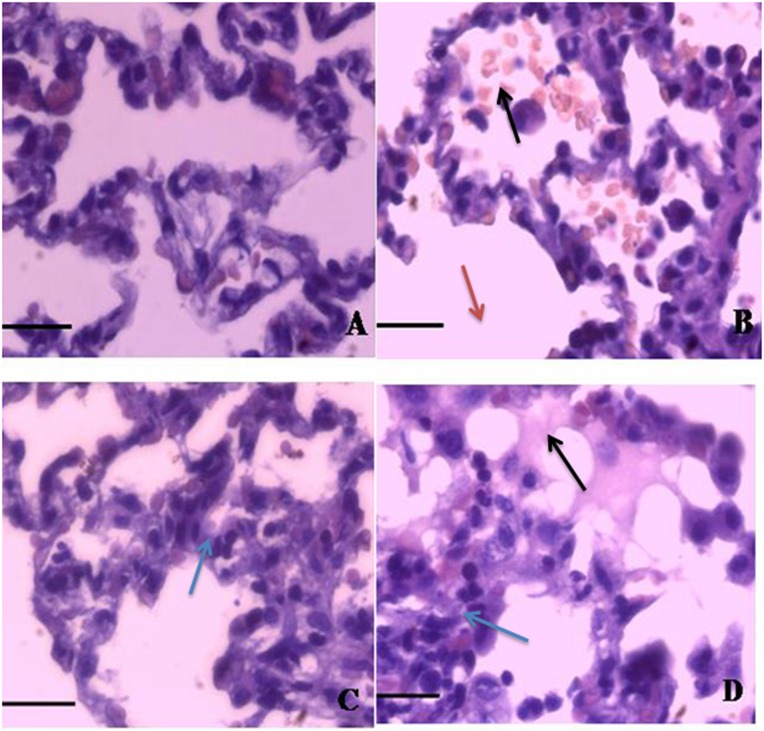
Microphotographs of representative lung section stained with hematoxylin and eosin (original magnification×400). A. The normal structure of lung from the control group (Group C); B. Severe alveolar edema, hemorrhage, the infiltration of leukocytes and thickened alveolar septum were observed in LPS group (Group L); C. Slight attenuation of the lung injury were displayed in treatment with electroacupuncture (Group EL); D. No improvement of the lung pathology were reflected in sham electroacupuncture stimulation (Group SEL). Black arrows: hemorrhage and infiltration of leukocytes in alveolar space; Red arrows: fracture of alveolar septum; Blue arrows: thickened alveolar septum. Scale bars: 50 µm.

Figure 1. illustrated the histopathological changes following LPS and the effects of electroacupuncture treatment at acupoints or non-acupoints. Administration of LPS gave rise to diffuse edema in alveolar spaces, infiltration and exudation of inflammatory cells into alveolar space, hemorrhage and thickened alveolar septum under light microscopy. In comparison, the morphological changes were far less pronounced with pretreatment of electroacupuncture. The scores of acute lung injury were summarized in Table 4. The levels of the lung injury scores were decreased in group EL compared with group L (P<0.05). However, in rabbits treated with sham electroacupuncture, the lung injury scores were similar with group L (P>0.05).

**Table 4:** Analysis of the histological assessment among four groups.

Groups	Acute lung injury scores
C	1(0–2)
L	10.5(8–14)*
EL	7.5(5–10)* +
SEL	11.5(7–15)*

Values were expressed as medians (range). The lung injury was scored by a 5-point scale according to combined assessments of alveolar congestion, hemorrhage and edema, infiltration or aggregation of neutrophils in the airspace or vessel wall and thickness of alveolar wall/hyaline membrane formation: Score of 0 = minimal (little) damage; 1+ = mild damage; 2+ = moderate damage; 3+ = severe damage; and 4+ = maximal damage. Minimum and maximum possible lung injury scores are 0 and 16, respectively.

*P<*0.05* versus Group C;

+ *P*<*0.05* versus Group L.

### The expressions of HO-1 mRNA, Nrf2 mRNA, HO-1 protein, Nrf2 total protein and Nrf2 nucleoprotein in lung tissue

**Figure 2:**
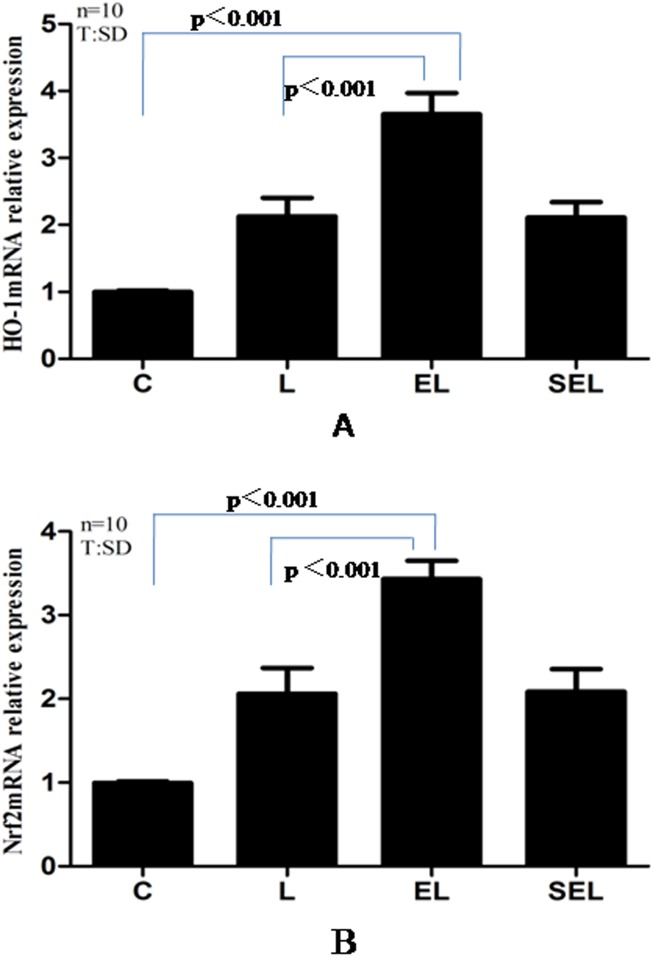
Real-time PCR analysis of HO-1 mRNA (A) and Nrf2 mRNA (B) expressions in the lung tissue of four groups. Data were presented as mean±SD. “C” presents group C, “L” presents group LPS, “EL” presents group electroacupuncture + LPS and “SEL” presents group sham electroacupuncture + LPS. The relative expressions of HO-1 mRNA and Nrf2 mRNA in group EL were higher than that in group C and group L (*P*<*0.05*), while no significant differences were found between group SEL and group L in terms of the above mentioned mRNA expressions(*P*>*0.05*). Ten control, LPS, EL and SEL experiments were performed for each group.

**Figure 3:**
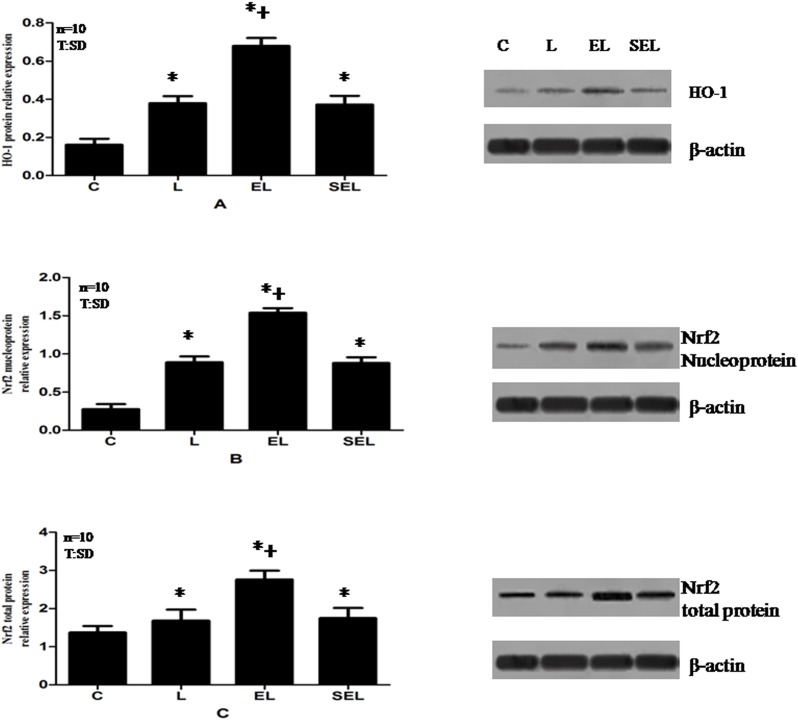
Western blot analysis of HO-1 protein (A), Nrf2 nucleoprotein (B) and Nrf2 total protein (C) relative expressions in lung tissue of four groups. Data were presented as mean±SD. “C” presents group C, “L” presents group LPS, “EL” presents group electroacupuncture + LPS and “SEL” presents group sham electroacupuncture + LPS. The expressions of HO-1 protein, Nrf2 nucleoprotein and total protein in group EL were higher compared with group C and group L (*P*<*0.05*). While sham electroacupuncture treatment exhibited the similar expressions of the above mentioned proteins to that of group L (*P*>*0.05*). Ten control, LPS, EL and SEL experiments were performed for each group. *P<0.05 versus control group;^ +^P<0.05 versus LPS group.

The results of the experiment determined by Real-time PCR and Western blot were shown in Figure 2 and Figure 3. Exposure to LPS notably increased the mRNA expression of HO-1 and Nrf2 as well as the protein expression of HO-1 and Nrf2 containing nucleoprotein and total protein compared with group C (P<0.05). In addition, the expression of HO-1 m RNA and Nrf2 mRNA plus the levels of HO-1 protein and Nrf2 total and nucleoprotein were markedly up-regulated in group EL in contrast to group L and group SEL (P<0.05). Nevertheless, there were no significant differences between group SEL and group L in terms of the above mentioned mRNA or protein expressions (P>0.05).

### The distribution ratio of Nrf2 nucleoprotein expression

**Figure 4:**
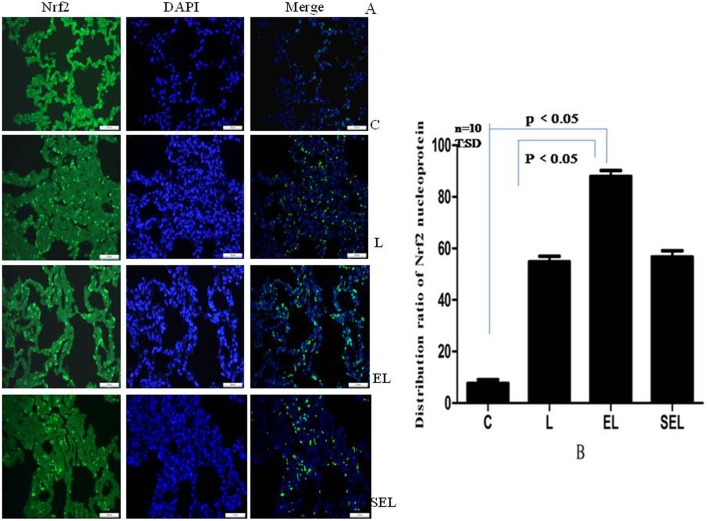
Immunofluorescence assays of nuclear localization of Nrf2 protein using fluorescence microscope (original magnification×400). “A” showed the pictures of immunofluorescence staining, while “B” presented the distribution ratios of Nrf2 nucleoprotein to the number of nuclei in unit area of five fields among four groups. Green standed for Nrf2-FITC stained sections, while blue standed for images of DAPI stained nuclei. It was confirmed that Nrf2 increasingly translocated from cytoplasm into the nucleus by electroacupuncture protocols (*P*<*0.05*) rather than sham electroacupuncture stimulation (*P*>*0.05*). Data were representative of three independent experiments. Values were mean ± SD, and ten control, LPS, EL and SEL experiments were performed for each group.

Immunofluorescence analysis of Nrf2 expression was represented in Figure 4. Group C showed a negligible Nrf2 nucleoprotein expression, while an enhanced expression with concomitant increase in Nrf2 positive protein was apparent in group L (P<0.05). Meanwhile, electroacupuncture stimulation at acupoints of ST36 and BL13 resulted in a significant increase in the number of Nrf2 nucleoprotein comparison with group L (augment 70.2%, P<0.05). However, sham electroacupuncture treatment exhibited the similar expression of Nrf2 to that of group L (P>0.05).

## Discussion

Data from the current study demonstrated that electrostimulation at ST36 and BL13 acupoints dramatically mitigated LPS-induced ALI in endotoxic shock rabbits. Furthermore, the production of HO-1 mRNA and HO-1 protein in group EL were notably higher than group L, which were consistent with the expression of Nrf2 mRNA, Nrf2 total protein and nucleoprotein. In addition, electroacupuncture treatment enhanced the activities of SOD, GPx and CAT with the increase of Nrf2 and following HO-1 expression. In brief, the present study for the first time confirmed that electroacupuncture at bilaterally ST36 and BL13 produced powerful protection against lung injury through activation of the Nrf2/ARE pathway and induction of the following antioxidant enzymes.

Systemic LPS exposure was used for establishing the standard model of endotoxic shock, which was invariably associated with ALI or ARDS and even multiorgan dysfunction [30]. ALI induced by endotoxin was manifested with hypoxemia and pulmonary edema depended on severe leukocytes infiltration, increased microvascular permeability and endothelial barrier disruption. The excess production of ROS by polymorphonuclear leucocytes exceeded the antioxidant defense capacity of cells and extracellular fluids, which leaded to oxidative damage in multiple organs [31]. MDA was a reliable marker of oxidative stress mediated lipid peroxidation [32]. Therefore, we applied it to reflect the degree of cell damage caused by reactive oxygen metabolites. The first line of defense against ROS mediated oxidative stress injury involved endogenous antioxidant enzymes such as SOD, GPx and CAT [4], [33]. Moreover, plasma levels of TNF-α and IL-6 were measured as indicators of systemic inflammatory responses [34], [35]. In our study, the activities of SOD, GPx and CAT were significantly decreased in LPS induced groups, accompanied with the increased MDA contents as well as the higher levels of TNF-α and IL-6. Concordant with previous studies [13], the rabbit model of injured lung induced by endotoxic shock in this research was defined by the lowered MAP<75% of the baseline values and oxygenation index (PaO_2_/FiO_2_) ≤300 mmHg.

HO-1 is highly inducible under conditions of ischemia/reperfusion injury or inflammatory cytokines and serves as one of the most prominent lines of defense of the cell against oxidative stress [36]. Previous research showed that hemin pretreatment with ulinastatin in endotoxin treated rats resulted in an improved response by upregulating HO-1 protein followed by increasing CO and restraining increased oxidative stress [37]. Furthermore, Takaki et al. indicated [12], oxidative stress was closely related to HO-1 expression, and the expression of HO-1 protein was increased in critically ill patients, especially those with severe sepsis or septic shock. To our knowledge, the parameters of electroacupuncture treatment including the frequency of EA were critical for producing prophylactic effects [38]. Therefore, acupuncture was performed with a disperse-dense wave with 2 Hz/15 Hz lasted 15 min for 5 days consecutively before the experiment in the current study [8], [19]. Data from our research revealed that electroacupuncture stimulation attenuated ALI induced by LPS in rabbits through upregulation of HO-1 and reduction of MDA content, W/D weight ratio and lung injury scores as well as augment of SOD activities, which was compatible with our prior study [14].

Nrf2/ARE signaling pathway is essential for upregulating the expression of numerous antioxidant genes in response to a wide array of stimuli, and also protecting the cell against oxidative stress and inflammation [39]. Under physiological conditions, Keap1 promoted cytosolic Nrf2 degradation via the Cul3-dependent ubiquitin proteasome pathway. When exposure to redox modulators, the reactive cysteine residues of Keap1 was modified, leading to Nrf2 translocation and accumulation in the nucleus. Subsequently, Nrf2 dimerized with small Maf or Jun proteins that binded to the ARE sequence in the promoter regions of phase II detoxification enzymes and antioxidant proteins, which were activated ultimately to protect cells from ROS generation [40]. As mentioned, SOD, GPx and CAT are directly involved in ROS scavenge, thus, are deemed as very important antioxidant enzymes. The SOD decomposes superoxide radicals and produces H_2_O_2_. And, H_2_O_2_ is subsequent removed to water by CAT in the peroxisomes or by GPx oxidizing GSH in the cytosol [10]. Consequently, the activities of above antioxidant enzymes are proportional to the Nrf2 expression. Moreover, Nrf2 had been clarified in Tsai PS et al. research to mediate upregulation of HO-1 by LPS in human monocytic cells [41]. Judging from the present study, upregulation of HO-1mRNA and protein by electroacupuncture in endotoxic shock rabbits was displayed in the lung tissue, which showed the same tendency with expression of Nrf2 mRNA as well as Nrf2 total and nucleoprotein, followed the increase of SOD, GPx and CAT activities. Consistent with Western blot and real-time PCR analyses, immunofluorescence staining identified increments in Nrf2 protein accumulation in the nucleus of cells subsequent to electroacupuncture stimulation.

Collectively, our research has several limitations. First of all, the experimental model of injured lung induced by endotoxic shock was established by intravenous LPS injection, which was extracted from the cell wall of Gram-negative bacteria. However, the infection of pathogenic bacteria was not the common cause in clinical patients with endotoxic shock. As a result, it is not easily for us to extrapolate our conclusions to the clinical setting. Secondly, lung hyper-permeability causing pulmonary edema was deemed as the main mechanism of ALI/ARDS [42]. Therefore, the determination of albumin content and leukocyte count in bronchoalveolar lavage fluid should be added in further exploration to more fully evaluate the effect of electroacupuncture on the impaired lung. Finally, the expression levels of Nrf2 including total protein and nucleoprotein in our present study were both increased significantly. The findings were in coincidence with the research of Chen et al. [43], which still need further exploration for a suitable explanation.

In summary, treatment with electroacupuncture at ST36 and BL13 acupoints alleviated ALI induced by LPS in rabbits, which was counted on activation of Nrf2/ARE pathway and up-regulation of HO-1 expression. In addition, several kinases and signaling pathways have been implicated in the activation of Nrf2/ARE pathway. It has been reported that the MAPK cascade, PI3K/AKT and PKC signaling pathways facilitate the dissociation of Nrf2 from Keap1 and thereby influence the Nrf2/ARE pathway [44]. Nevertheless, the accurate mechanism by which electroacupuncture activates Nrf2 pathway has not been fully understood and requires further investigation. Above all, the present findings provide the scientific foundation for the development of electroacupuncture as a prophylactic treatment for acute lung injury induced by endotoxic shock in clinic.
